# Acute cholecystitis: a new technique to use carefully

**DOI:** 10.1186/s13017-025-00662-y

**Published:** 2025-12-16

**Authors:** Adriana Toro, Martina Rapisarda, Davide Maugeri, Alessandro Terrasi, Luisa Gallo, Luca Ansaloni, Fausto Catena, Isidoro Di Carlo

**Affiliations:** 1 Department of General Surgery and Medical Surgical Specialties, University of Catania, Via Santa Sofia 78, 95100 Catania, Italy; 2 Department of Surgical Sciences and Advanced Technologies “G.F. Ingrassia”, Cannizzaro Hospital, General Surgery, University of Catania, Catania, Italy; 3 Department of Clinical, Diagnostic and Pediatric Sciences, University of Pavia, Pavia, Italy; 4 General and Emergency Surgery, Bufalini Hospital, Cesena, Italy

## Abstract

This manuscript responds to a commentary published in the World Journal of Emergency Surgery in 2025;20:9 in which the authors criticized a new technique for the treatment of acute cholecystitis. Therefore, the authors of the manuscript published in the World Journal of Emergency Surgery in 2024;19:6, titled "Acute Cholecystitis: How to Avoid Subtotal Cholecystectomy—Preliminary Results," provide a critical point-by-point response and explain why this technique represents a new addition to the surgeon's armamentarium for very severe cholecystitis.

Dear editor,

I thank Dr. Pesce et al. for their attention to our new preliminary experience with a new technique for total cholecystitis in acute cholecystitis publish on World Journal of Emergency Surgery with title "Acute cholecystitis: how to avoid subtotal cholecystectomy-preliminary results" [1]. This new technique permits to separate adhesions of the gallbladder and makes an incision between the infundibulum and the body of the gallbladder. In this way there is a separation of planes between the outer layer (serosa) and the inner layer (muscular layer) up to the origin of the cystic duct. This step allows identification of the point of confluence between the cystic duct and the body of the gallbladder, avoiding damage to the common bile duct.

The cholecystectomy is performed cautiously as usual, leaving the inflamed posterior wall adherent to the liver bed.

Pesce et al. reported that three patients constituted a very small sample size for trauma center services [2]. This technique avoids all complications related to other methods reported in the literature. However, a new technique always presupposes the certainty of avoiding complications and therefore must be performed with the maximum of safety.

The 2018 Tokyo guidelines for cases of grade II acute state that early laparoscopic procedures could be indicated if advanced laparoscopic techniques are available and surgeons are skilled [3]. We report that the use of a single operator casuistic (IDC) in hepatobiliary surgery is one of the reasons for this initial experience, which must be safe for the patient and the surgeon.

The letter criticizes the use of three trocars to perform the surgical procedure. The use of three trocars to perform cholecystectomy was previously proposed in 1995, with the possibility of inserting a fourth trocar, if necessary, in cases of acute cholecystitis [4]. Three trocars can be used and insert the fourth trocar, if necessary, at any time. This obviously does not compromise the safety of the patient and does not expose the surgeon to medico-legal problems.

The cystic duct is secured by clip only if it is absolutely isolated, and it is the only structure present. An endostapler is not necessary for a gallbladder inflamed by this technique. The first rule for this technique is that the structure must be isolated and identified carefully. This approach avoids any risk for the adjacent structures and allows total cholecystectomies to be performed.

The technique described makes it possible to isolate the two layers that make up the wall of the gallbladder, the outer layer (serosa) and the inner layer (muscularis layer). This allows the cystic duct entering the infundibulum to be clearly identified. This technique enables the isolation and ligation of the cystic duct without risk. If this maneuver is not performed, 5-mm clips may not securely close the cystic duct because of the inflammatory condition of the tissue, potentially leading to postoperative cystic duct leakage.

Gangrenous cholecystitis, of course, cannot be treated with this technique.

However, we have reported three cases performed with this technique; therefore, it is not a theoretical hypothesis, and its feasibility is related to an experienced operator.

Obviously, larger studies are needed to confirm its validity, but our goal is to present a new technique that could be considered by surgeons in cases of acute cholecystitis [1].

Acute cholecystitis is characterized by intense inflammation, which causes edema of the outer layer (serosa). This allows a surgeon to use the electric hook delicately without involving the deeper layer. The use of scissors instead of an electric hook, due to the high degree of inflammation, may cause bleeding that can dirty the operating field and make dissection more difficult.

During laparoscopic cholecystectomy for acute cholecystitis, examination of the anatomy is mandatory, and a complete knowledge of the vascular anatomy of the gallbladder with inflammation is needed.

Our technique can be used in all cases of acute cholecystitis in which the inflammatory wall allows the identification of a cleavage plane between the internal and external layers. All our patients had stage II acute cholecystitis according to the Tokyo guidelines. However, with our technique, it was possible to identify it without any doubt. Therefore, sectioning of the cystic duct was performed without the risk of injury to the bile duct. As also reported by Strasberg with the critical view of safety [5], 2 structures, and only 2, should be seen entering the gallbladder. With our technique, it is possible to identify the cystic duct without any doubt.

Nassar described the use of subserosal dissection in thick-walled gallbladders when the gallbladder is adherent to the duodenum or the lateral wall of the bile duct. However, the article does not describe the cases in which this surgical procedure was performed. Therefore, it is not justified to state that this technique is not new [6].

Certainly, the first to analyze the various techniques of laparoscopic subtotal cholecystectomy was Strasberg (1995), but the classification into four types (A, B, C, D) draws inspiration from the vastness of techniques reported in the literature with the intent to clarify which is the best technique to reduce postoperative complications [7].

To date, the use of indocyanine green is not considered mandatory in difficult cholecystectomies, so its use remains at the discretion of the surgeon because it has not been proven to reduce bile duct injury, postoperative complications, or operative time. [8, 9].

Currently grade II acute cholecystitis is not managed by highly experienced surgeons, but rather by residents. In these cases, the new technique should be performed only if an experienced surgeon can help the resident. Otherwise, it would be preferable to switch to subtotal cholecystectomy, which represents a safe and reliable option for any surgeon.

Total cholecystectomy remains the optimal procedure because it avoids the postoperative complications of subtotal cholecystectomy, such as biliary fistula, subhepatic collection, abdominal infection and residual stones (10, 11).

This technique represents a new technique in the armamentarium of surgeons in cases of very difficult cholecystitis. This is not a “panacea” to solve all problems related to this surgical procedure, but it can be useful if adverse conditions persist.

## Data Availability

No datasets were generated or analysed during the current study.
